# Multicenter evaluation of the multiplex polymerase chain reaction QIAstat-Dx Meningitis/Encephalitis Panel for the detection of central nervous system pathogens directly in the cerebrospinal fluid

**DOI:** 10.1128/jcm.00738-26

**Published:** 2026-10-27

**Authors:** Christopher Farnsworth, Ping Ren, Juan José González-López, Johanna Bialas, Romney Humphries, Thomas Davis, Patricia J. Simner, Nathan A. Ledeboer, James Dunn, Neelam Dhiman, Yvan Caspar, Donna M. Wolk, Melisa Rey, Sarah Fern, Alistair English, Sarah Johnson, Preeti Pancholi

**Affiliations:** 1 Department of Pathology and Immunology, WUSTL 12299, St. Louis, Missouri, USA; 2 University of Texas Medical Branch Health 595024, Galveston, Texas, USA; 3 University of Illinois Chicago, Chicago, Illinois, USA; 4 Vall d’Hebron University Hospital 16810 , Barcelona, Spain; 5 CIBER en Enfermedades Infecciosas (CIBERINFEC), Instituto de Salud Carlos III 38176, Madrid, Spain; 6 Labor Berlin Services, Berlin, Germany; 7 Vanderbilt University Medical Center 12328, Nashville, Tennessee, USA; 8 Indiana University School of Medicine 12250, Indianapolis, Indiana, USA; 9 Johns Hopkins University School of Medicine 1500, Baltimore, Maryland, USA; 10 Department of Laboratory Medicine and Pathology, Mayo Clinic 195112, Rochester, Minnesota, USA; 11 Medical College of Wisconsin 5506, Milwaukee, Wisconsin, USA; 12 Baylor College of Medicine, Texas Children’s Hospital 3989, Houston, Texas, USA; 13 Quest Diagnostics 2780 , Lewisville, Texas, USA; 14 University Grenoble Alpes, Grenoble, France; 15 CNRS, Grenoble, France; 16 CEA, Grenoble, France; 17 IBS 55543 , Grenoble, France; 18 CHU Grenoble Alpes 36724 , Grenoble, France; 19 Geisinger Diagnostic Medicine Institute, Danville, Pennsylvania, USA; 20 STAT-Dx Life S.L. (a QIAGEN company), Barcelona, Spain; 21 QIAGEN Ltd. 118818 , Manchester, England, United Kingdom; 22 The Ohio State University Wexner Medical Center 12306, Columbus, Ohio, USA; Marquette University, Milwaukee, Wisconsin, USA

**Keywords:** multiplex, molecular diagnostics, encephalitis, QIAstat Dx, meningitis

## Abstract

Rapid and accurate detection of pathogens causing meningitis/encephalitis (MEN/EN) is critical to improving clinical outcomes. Multiplex polymerase chain reaction (PCR) enables simultaneous detection of multiple pathogens from the cerebrospinal fluid (CSF), offering advantages over traditional methods. This multicenter study evaluated the performance of the QIAstat-Dx Meningitis/Encephalitis (ME) Panel versus conventional methods, including the BioFire FilmArray ME (BFME) Panel. The CSF samples from patients with suspected MEN/EN along with contrived samples were tested across 13 sites in Europe/USA. Positive percent agreement (PPA) and negative percent agreement (NPA) between the QIAstat-Dx ME Panel and comparator methods were determined. A total of 1,667 clinical samples (1,527 prospective, 140 archived) were tested. Pathogens were detected by the QIAstat-Dx ME Panel in 115 (7.5%) prospective samples, including *Streptococcus pneumoniae* (1.1%), *Haemophilus influenzae* (0.5%), *Listeria monocytogenes* (0.3%), *S. agalactiae* (0.2%), *Escherichia coli* K1 (0.1%), *Neisseria meningitidis* (0.1%), *S. pyogenes* (0.1%), enterovirus (1.5%), human herpesvirus-6 (1.0%), herpes simplex virus-2 (0.9%) and −1 (0.3%), varicella zoster virus (0.7%), cytomegalovirus (0.3%), human parechovirus (0.3%), and *Cryptococcus gattii/neoformans* (0.4%). Overall PPA versus BFME (*n* = 1,664) was 85.4%, increasing to 95.3% after discordance resolution. PPA was 100% for *N. meningitidis*, *S. pneumoniae*, and *S. agalactiae*. Overall NPA was 99.9%. Among 1,154 contrived positive samples, only 27 (2.3%) incorrect results were observed. The QIAstat-Dx ME Panel demonstrated high concordance with BFME, supporting its use as a reliable, fully automated multiplex PCR platform for rapid diagnosis of community-acquired MEN/EN.

**IMPORTANCE:**

This multicenter study demonstrated that the QIAstat-Dx ME Panel delivers clinical performance comparable to that of the BioFire FilmArray ME (BFME) Panel, supporting its validity as a reliable syndromic molecular diagnostic tool for MEN/EN. With its broad pathogen coverage, rapid turnaround time (TAT), and the ability to provide amplification curves and Ct values, the QIAstat-Dx ME Panel offers a valuable option for broad and timely detection of bacterial, viral, and fungal CNS pathogens. When used alongside the SoC diagnostic methods, this panel has the potential to improve early diagnosis, guide targeted therapy, and support effective clinical management of patients with suspected MEN/EN.

## INTRODUCTION

Meningitis and encephalitis (MEN/EN) are severe inflammatory conditions affecting the central nervous system (CNS). MEN involves inflammation of the meninges, the membranes surrounding the brain and the spinal cord, while encephalitis affects the brain parenchyma itself (1, 2). Both conditions may be caused by infectious agents (bacteria, viruses, or fungi) or non-infectious conditions, such as autoimmune or neoplastic diseases, and they typically present with headaches, fever, and altered mental status (2–4). Bacterial MEN can progress rapidly and is often fatal without prompt treatment, while both syndromes may result in long-term neurological complications (2, 3). Despite improvements in supportive care, morbidity and mortality remain high (1, 2), making early diagnosis and timely initiation of appropriate therapy critical to improving outcomes (4).

The etiologic spectrum of MEN/EN varies by patient population and geographic region (2, 5), but infectious agents are frequently responsible for the most severe cases (5). Although vaccination has reduced the incidence of some forms of MEN, the global burden remains high, with over 2.5 million cases reported annually (1). The specific etiology remains unidentified in approximately 40% of patients with MEN (6) and up to 75% of EN cases (7), highlighting the urgent need for rapid, sensitive, accurate, and accessible diagnostic tools. Standard diagnostic workflows involve cerebrospinal fluid (CSF) collection via lumbar puncture, followed by Gram stain, culture, cryptococcal antigen detection, and pathogen-specific polymerase chain reaction (PCR) (4, 8). CSF culture, the reference (gold) standard for bacteria, is limited by sensitivity, long turnaround times (TAT; up to 5 days), potential interference from prior antibiotic exposure, the requirement for larger sample volumes, and the risk of missing fastidious or slow-growing organisms (9). Although diagnostic algorithms have been designed to address some of these limitations (8, 10), the need for rapid, sensitive, and broad syndromic testing remains (9).

Multiplex PCR addresses many of these challenges by enabling simultaneous detection of multiple pathogens in a single test, with greater speed and sensitivity than traditional methods; for example, the BioFire FilmArray Meningitis/Encephalitis (ME) Panel (BFME; bioMérieux, Marcy l’Etoile, France) approved by the United States (U.S.) Food and Drug Administration (FDA) in 2015 detects 14 pathogens (seven viruses, six bacteria, and one fungus) in under 1 h and has gained widespread clinical adoption (11–13). Its use is now recommended in guidelines for community-acquired MEN (14) and has been shown to support targeted therapy and reduce unnecessary antimicrobial use (11, 12).

The QIAstat-Dx Meningitis/Encephalitis (ME) Panel (QIAGEN, Germany) is a fully automated multiplex PCR system that detects 16 pathogens associated with community-acquired MEN/EN, including eight bacteria, seven viruses, and one fungal target, directly from CSF, in approximately 80 min (15, 16). In addition to qualitative results, the panel provides cycle threshold (Ct) values and amplification curves to aid interpretation. Targets include *Escherichia coli* K1, *Haemophilus influenzae*, *Listeria monocytogenes*, *Mycoplasma pneumoniae*, *Neisseria meningitidis* (encapsulated), *Streptococcus agalactiae*, *S. pneumoniae*, *S. pyogenes*, herpes simplex virus-1 and -2 (HSV-1 and -2), human herpesvirus-6 (HHV-6), enterovirus (EV), human parechovirus (HPeV), varicella zoster virus (VZV), cytomegalovirus (CMV), and *Cryptococcus neoformans/Cryptococcus gattii* (not differentiated). The panel is Conformité Européenne—*In Vitro* Diagnostic Device Regulation (CE-IVDR) marked (15). In the USA, a version of the panel, including nine bacterial, viral, and fungal targets, is available. The CE-IVD configuration additionally includes CMV, HSV-1, HSV-2, HHV-6, HPeV, and VZV. The inclusion of these viral targets expands coverage to clinically relevant causes of meningitis and encephalitis across diverse patient populations, including neonates, infants, immunocompromised individuals, and patients with viral encephalitis and/or meningitis.

This multicenter study evaluated the performance of the CE-IVDR-marked QIAstat-Dx ME Panel using residual CSF samples from patients with suspected MEN/EN. Performance was compared with established reference and comparator methods, including the BFME Panel, for detection of 16 clinically relevant pathogens.

## MATERIALS AND METHODS

### Study design

**Fig 1:**
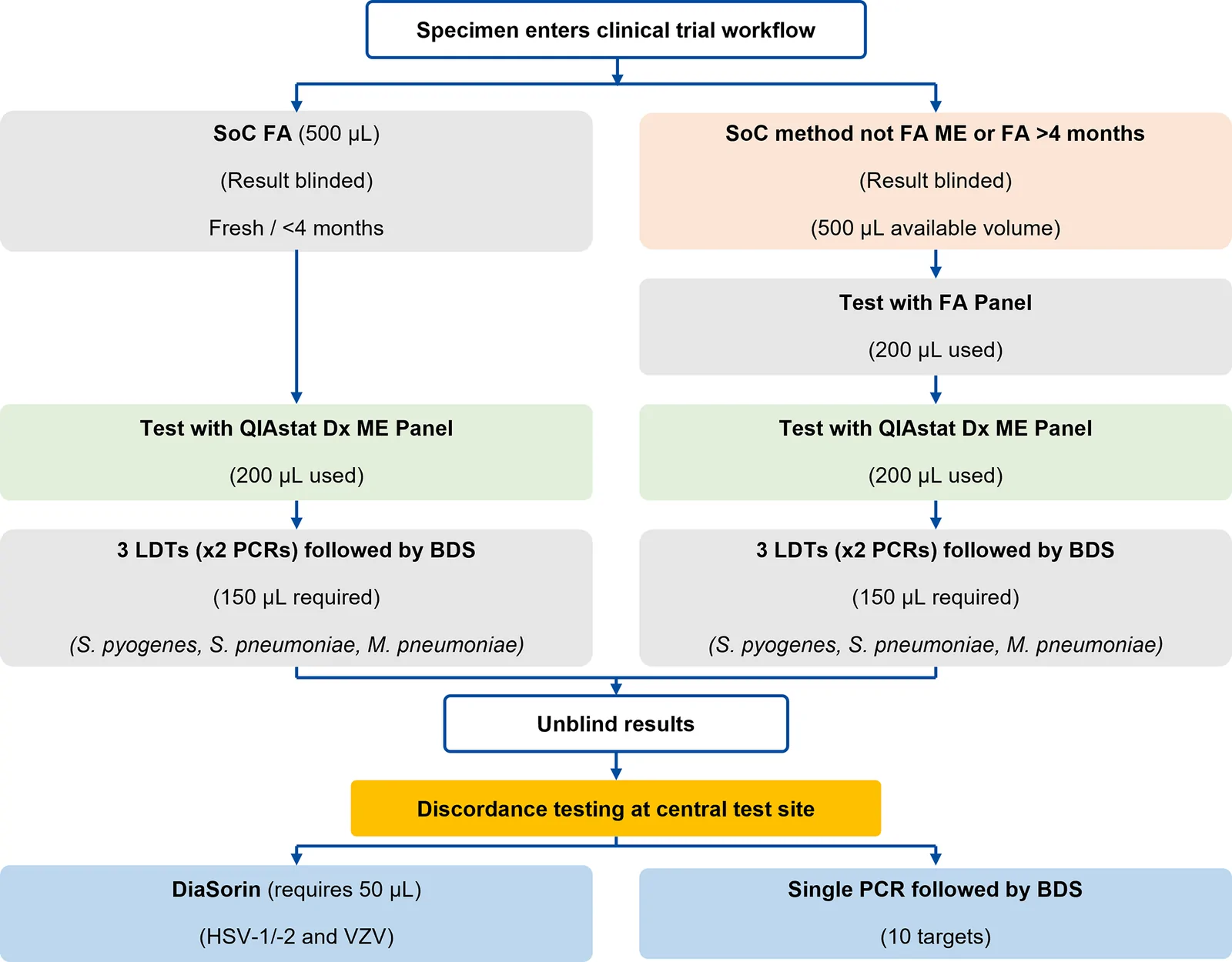
Specimen processing workflow. BDS, bidirectional sequencing; FA, BioFire FilmArray ME Panel; HSV, herpes simplex virus; LDT, laboratory-developed test; PCR, polymerase chain reaction; SoC, standard of care; and VZV, varicella zoster virus. Following comparator testing, all samples were frozen and stored before being shipped to central testing sites for additional comparator testing or discordance testing if required.

This multicenter method comparison study was conducted at 13 sites across four countries (France, Germany, Spain, and the USA) between 8 September 2021 and 2 March 2023. The study evaluated the performance of the QIAstat-Dx ME Panel for detecting pathogens in patients with clinical signs and symptoms of MEN/EN. Residual fresh and frozen CSF samples were tested and compared against established reference methods. The specimen processing workflow is shown in Fig. 1.

### Endpoints

The primary endpoint of the study was to determine the positive percent agreement (PPA) and negative percent agreement (NPA) of the QIAstat-Dx ME Panel with comparator methods.

### Sample collection and preparation

Residual, de-identified, unique CSF samples were obtained via lumbar puncture from patients of all ages with clinical suspicion of MEN/EN and with a minimum residual volume of 500 µL. The samples were grouped as: (i) prospective fresh (tested without freezing); (ii) prospective frozen (collected fresh but frozen before testing); and (iii) archived (previously banked, known-positive frozen samples). Samples were stored at room temperature for ≤24 h, refrigerated at 2–8°C for ≤7 days, or frozen at ≤−70°C (± 20°C) prior to QIAstat-Dx ME Panel testing. Frozen samples tested with the QIAstat-Dx ME Panel underwent no more than three freeze–thaw cycles in accordance with established specimen stability criteria. A dedicated PCR stability study demonstrated no detrimental effect on target detection following up to five freeze–thaw cycles for representative organisms. Both testing approaches were, therefore, performed under storage and handling conditions supported by their respective stability studies. A minimum of 1,500 prospective samples was required per FDA guidance for multiplex assay evaluation (17).

### Contrived samples

Contrived samples were used to supplement testing for targets with insufficient positive clinical samples. QIAGEN Manchester Ltd. (Manchester, UK) spiked known pathogen concentrations into individual PCR-negative CSF samples tested alongside clinical samples. Over 50% of contrived samples were near the assay limit of detection (LoD; i.e., 2 × LoD), with the remainder at 5 × LoD. A minimum of five different analytical strains per pathogen were used to represent genetic diversity per regulatory guidance.

Bulk CSF stored at −60 to −90°C was used for LoD determination, defined as the lowest concentration with >95% detection in replicates (data not shown).

### Blinding and bias mitigation

Operators were blinded to standard-of-care (SoC) results. Contrived samples were blinded and mixed with negative samples. Additional contrived samples were tested after completion of prospective sample collection.

### Sample handling

All samples were labeled with unique study identifiers. Frozen samples were tested within 90 min of thawing. After completion of BFME and QIAstat-Dx ME Panel testing, samples were frozen at −70°C (± 20°C) and shipped to central testing sites for additional comparator or discordance testing as needed.

### Testing procedures

The QIAstat-Dx ME Panel, a fully automated, real-time multiplex PCR platform, was used per manufacturer’s instructions (see Supplementary Appendix for summary of methodology) (15). Pathogen targets are listed in Table S1. Daily quality control included three rotating pathogen-positive mixes and one pathogen-negative external control mix (composition shown in Table S2).

Comparator methods included the BFME for all targets, except *S. pyogenes* and *M. pneumoniae*, which are not included in this panel, and *S. pneumoniae*, due to previously reported high false-positive rates with the BFME (9, 11, 18, 19). The validation study in BFME instructions for use identified four true positives and 12 apparent false positives, such that if BFME was used as a comparator, the highest PPA a new test could achieve would be 25%. For these three pathogens, endpoint PCR assays developed and validated by Eurofins Genomics (Ebersberg, Germany) were used, with analysis performed on the LightCycler 480 Instrument II (Roche Diagnostics GmbH, Mannheim, Germany) or the Veriti Dx 96-Well Thermal Cycler (Applied Biosystems, Waltham, Massachusetts, USA). Bidirectional sequencing (BDS) was performed for positive results by Eurofins Genomics. For *S. pyogenes*, two PCR assays were used with amplicon sizes of 259 and 215 bp. For *M. pneumoniae*, two PCR assays with amplicon sizes of 310 and 308 were used, and for *S. pneumoniae*, two PCR assays with amplicon sizes of 400 and 305 bp were used. Samples were processed in triplicate. All PCR assays were designed to be at least as sensitive as the Limit of Detection (LoD) of the corresponding QIAstat-Dx ME Panel. This was confirmed for each assay by a hit rate of ≥95% when testing 20 samples at the corresponding QIAstat-Dx ME Panel LoD, except for *M. pneumoniae* where the LoD was established at 9.99 E + 02 CFU/mL.

### Discordant result investigation

Discordant results were resolved when sufficient sample volume remained. Methods included culture from the SoC, validated PCR with BDS by Eurofins Genomics, or Simplexa HSV 1 & 2 and VZV Direct Kits (DiaSorin, Saluggia, Italy) for herpes viruses (20, 21).

### Statistical analysis

Primary analysis included only clinical CSF samples with valid results from both the QIAstat-Dx ME Panel and comparator methods. Concordance was classified as true positive (TP), true negative (TN), false positive (FP), or false negative (FN). PPA was calculated as TP/(TP + FN) and NPA as TN/(TN + FP). Proportions were reported with 95% two-sided Wilson-score confidence intervals (CI) (11).

## RESULTS

### Sample collection

**Fig 2:**
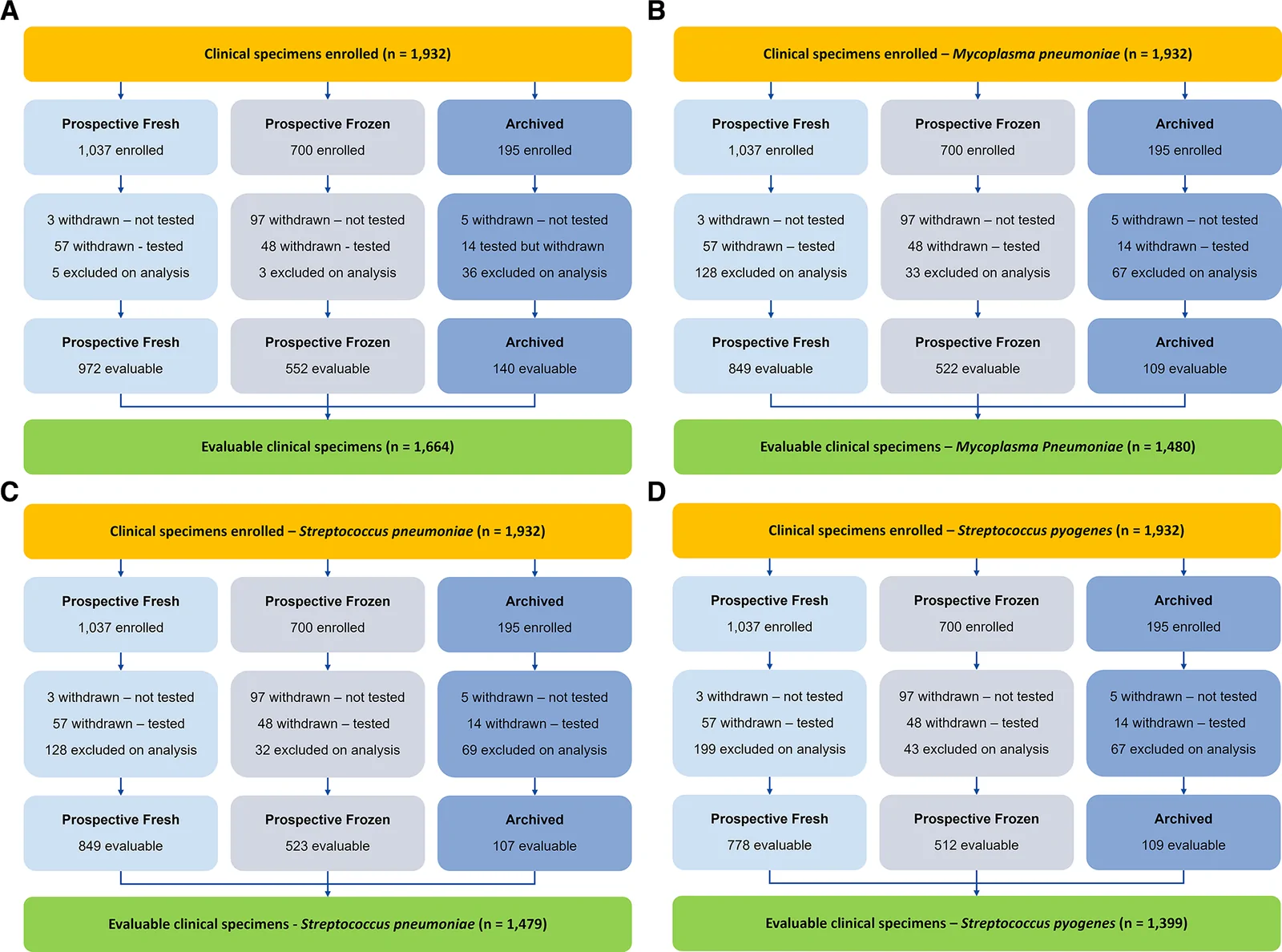
Sample disposition for all target pathogens when the comparator method was BioFire FilmArray Panel (**A**) and *M. pneumoniae*, *S. pneumoniae*, and *S. pyogenes* when the comparator method was PCR-BDS (**B–D**). PCR-BDS, polymerase chain reaction with bidirectional sequencing.

A total of 1,932 clinical CSF samples were collected. Of these, 224 (11.6%) were withdrawn or excluded due to incorrect testing workflow, sample ineligibility, or testing deviation from the protocol. An additional 44 samples were excluded from the primary analysis due to failed runs, absence of comparator results, or, for archived “known positive” samples, lack of confirmation of the expected result on the applicable comparator method. This resulted in 1,664 evaluable clinical samples for PPA/NPA analysis by the BFME Panel comprising 1,524 prospectively collected and 140 archived samples (Fig. 2A). For *M. pneumoniae*, *S. pneumoniae*, and *S. pyogenes*, comparator testing with PCR and BDS was conducted on 1,480, 1,479, and 1,399 clinical samples, respectively (Fig. 2B through D). Demographic data of the samples are summarized in Table 1.

**TABLE 1:** Demographic characteristics of all BFME Panel samples included in the study

Archived (*n* = 140)	*n* (%)
Age group, years	
<1	13 (9.3)
1–17	14 (10.0)
18–44	34 (24.3)
45–64	32 (22.9)
65–84	39 (27.9)
≥85	8 (5.7)
Sex	
Female	78 (55.7)
Male	62 (44.3)

Prospective fresh (*n* = 972)	*n* (%)
Age group, years	
<1	136 (14.0)
1–17	87 (9.0)
18–44	284 (29.2)
45–64	266 (27.4)
65–84	187 (19.2)
≥85	11 (1.1)
Unknown	1 (0.1)
Sex	
Female	498 (51.2)
Male	474 (48.8)

Prospective frozen (*n* = 552)	*n* (%)
Age group, years	
<1	27 (4.9)
1–17	41 (7.4)
18–44	133 (24.1)
45–64	174 (31.5)
65–84	156 (28.3)
≥85	20 (3.6)
Unknown	1 (0.2)
Sex	
Female	271 (49.1)
Male	280 (50.7)
Unknown	1 (0.1)

To meet the minimum threshold of 30 positives per target, 1,154 contrived positive samples were prepared: *E. coli* K1 (*n* = 85), *H. influenzae* (*n* = 93), *L. monocytogenes* (*n* = 87), *M. pneumoniae* (*n* = 86), *N. meningitidis* (*n* = 88), *S. agalactiae* (*n* = 88), *S. pneumoniae* (*n* = 96), *S. pyogenes* (*n* = 89), CMV (*n* = 89), EV (*n* = 88), HSV-1 (*n* = 99), HPeV (*n* = 87), and *C. gattii/neoformans* (*n* = 79).

### QIAstat-Dx ME positivity rates

Positivity was based on prospective clinical samples with a valid QIAstat-Dx ME result, comprising 1,524 samples from the BFME primary analysis plus three additional samples without a valid BFME result. Overall, 115 samples (7.5%) were positive for at least one pathogen. Bacterial positives included: *S. pneumoniae* (*n* = 17 [1.1%]), *H. influenzae* (*n* = 7 [0.5%]), *L. monocytogenes* (*n* = 4 [0.3%]), *S. agalactiae* (*n* = 3 [0.2%]), *E. coli* K1 (*n* = 2 [0.1%]), *N. meningitidis* (*n* = 2 [0.1%]), and *S. pyogenes* (*n* = 1 [0.1%]). Viral positives included: EV (*n* = 23 [1.5%]), HHV-6 (*n* = 15 [1.0%]), HSV-2 (*n* = 14 [0.9%]), VZV (*n* = 10 [0.7%]), CMV (*n* = 5 [0.3%]), HPeV (*n* = 5 [0.3%]), and HSV-1 (*n* = 4 [0.3%]). The fungal target *C. gattii*/*neoformans* was detected in six (0.4%) samples. Archived samples were not included in determining the prevalence to ensure data are representative of the intended use population.

### Agreement with comparator methods

Among clinical samples with valid comparator results (*n* = 1,664), the QIAstat-Dx ME Panel demonstrated an overall PPA of 85.4% (95% confidence interval [CI]: 80.6–89.2%) and an NPA of 99.9% (95% CI: 99.9–99.9%). Three bacterial targets achieved 100.0% PPA: *N. meningitidis* (4/4), *S. agalactiae* (12/12), and *S. pneumoniae* (12/12; Table 2). Ct values for all detected pathogens are shown in Fig. S1. *M. pneumoniae* and *S. pyogenes* not covered by BFME were not detected by the QIAstat-Dx ME Panel in any clinical samples with a valid comparator result.

**TABLE 2:** PPA and NPA between the QIAstat-Dx ME Panel and comparator method for all clinical samples by target before resolution testing^*a*^

	PPA^*b*^			NPA^*b*^		
	TP/TP + FN	%	95% CI	TN/TN + FP	%	95% CI
All pathogens	222/260	85.4	80.6–89.2	25,712/25,736	99.9	99.9–99.9
Bacteria						
*E. coli* K1	4/6	66.7	30.0–90.3	1,658/1,658	100	99.8–100
*H. influenzae*	10/11	90.9	62.3–98.4	1,650/1,653	99.8	99.5–99.9
*L. monocytogenes*	4/5	80.0	37.6–96.4	1,659/1,659	100	99.8–100
*M. pneumoniae*	0/0	N/A	N/A	1,482/1,482	100	99.7–100
*N. meningitidis*	4/4	100	51.0–100	1,659/1,660	99.9	99.7–100
*S. agalactiae*	12/12	100	75.8–100	1,652/1,652	100	99.8–100
*S. pneumoniae*	12/12	100	75.8–100	1,463/1,469	99.6	99.1–99.8
*S. pyogenes*	0/0	N/A	N/A	1,401/1,401	100	99.7–100
Overall	46/50	92.0	81.2–96.8	12,624/12,634	99.9	99.9–100
Viruses						
CMV	3/5	60.0	23.1–88.2	1,656/1,659	99.8	99.5–99.9
EV	31/33	93.9	80.4–98.3	1,630/1,631	99.9	99.7–100
HSV-1	10/12	83.3	55.2–95.3	1,652/1,652	100	99.8–100
HSV-2	29/36	80.6	65.0–90.2	1,627/1,628	99.9	99.7–100
HPeV	4/8	50.0	21.5–78.5	1,655/1,656	99.9	99.7–100
HHV-6	25/30	83.3	66.4–92.7	1,628/1,634	99.6	99.2–99.8
VZV	62/71	87.3	77.6–93.2	1,593/1,593	100.0	99.8–100.0
Overall	164/195	84.1	78.3–88.6	11,441/11,453	99.9	99.8–99.9
Fungi & yeast						
*C. gattii / C. neoformans* (not differentiated)	12/15	80.0	54.8–93.0	1,647/1,649	99.9	99.6–100.0

^*a*^ CI, confidence interval; CMV, cytomegalovirus; EV, enterovirus; FN, false negative; FP, false positive; HHV, human herpesvirus; HPeV, human parechovirus; HSV, herpes simplex virus; N/A, not applicable; NPA, negative percent agreement; PPA, positive percent agreement; TN, true negative; TP, true positive; VZV, varicella zoster virus.

^*b*^ Data shown are for positive samples with a valid comparator result.

### Contrived samples

All contrived samples were tested at 2 and 5 × the assay LoD. Among 1,154 positive contrived samples, the detection rate ranged from 92.0 to 100.0% across targets (Table 3), with 27 false positives. Among 676 negative contrived samples, 99.7–100.0% were negative, with four false positives: two *N. meningitidis*, one EV, and one *C. gattii*/*neoformans*.

**TABLE 3:** Overall proportion of positive results for contrived positive samples for each target^*a*^

Pathogen	Concentration level	Frequency of positive results^*b*^	Proportion of positive results (%)	95% CI
Bacteria				
*E. coli* K1	2 × LoD	48/48	100	92.6–100
	5 × LoD	37/37	100	90.6–100
	Total	85/85	100	95.7–100
*H. influenzae*	2 × LoD	57/57	100	93.7–100
	5 × LoD	36/36	100	90.4–100
	Total	93/93	100	96.0–100
*L. monocytogenes*	2 × LoD	47/49	95.9	86.3–98.9
	5 × LoD	38/38	100	90.8–100
	Total	85/87	97.7	92.0–99.4
*M. pneumoniae*	2 × LoD	46/46	100	92.3–100
	5 × LoD	39/40	97.5	87.1–99.6
	Total	85/86	98.8	93.7–99.8
*N. meningitidis*	2 × LoD	46/48	95.8	86.0–98.8
	5 × LoD	39/40	97.5	87.1–99.6
	Total	85/88	96.6	90.5–98.8
*S. agalactiae*	2 × LoD	49/49	100	92.7–100
	5 × LoD	39/39	100	91.0–100
	Total	88/88	100	95.8–100
*S. pneumoniae*	2 × LoD	55/57	96.5	88.1–99.0
	5 × LoD	39/39	100	91.0–100
	Total	94/96	97.9	92.7–99.4
*S. pyogenes*	2 × LoD	47/49	95.9	86.3–98.9
	5 × LoD	40/40	100	91.2–100
	Total	87/89	97.8	92.2–99.4
Viruses				
CMV	2 × LoD	46/50	92.0	81.2–96.8
	5 × LoD	39/39	100.0	91.0–100.0
	Total	85/89	95.5	89.0–98.2
EV	2 × LoD	48/49	98.0	89.3–99.6
	5 × LoD	39/39	100.0	91.0–100.0
	Total	87/88	98.9	93.8–99.8
HSV-1	2 × LoD	50/52	96.2	87.0–98.9
	5 × LoD	45/47	95.7	85.8–98.8
	Total	95/99	96.0	90.1–98.4
HPeV	2 × LoD	46/48	95.8	86.0–98.8
	5 × LoD	39/39	100.0	91.0–100.0
	Total	85/87	97.7	92.0–99.4
Fungi and yeast				
*C. gattii* / *C. neoformans* (not differentiated)	2 × LoD	41/41	100.0	91.4–100.0
	5 × LoD	38/38	100.0	90.8–100.0
	Total	79/79	100.0	95.4–100.0

^*a*^ CI, confidence interval; CMV, cytomegalovirus; EV, enterovirus; HPeV, human parechovirus; HSV, herpes simplex virus; LoD, limit of detection.

^*b*^ False positives were detected for the following pathogens: *S. pneumoniae* (*n* = 7), *M. pneumoniae* (*n* = 4), *L. monocytogenes* (*n* = 3), *S. agalactiae* (*n* = 2), *E. coli* K1 (*n* = 2), *N. meningitidis* (*n* = 1), HPeV (*n* = 2), CMV (*n* = 2), EV (*n* = 1), HSV-1 (*n* = 1), and *C. gattii* / *C. neoformans* (not differentiated) (*n* = 2).

### Discordant result investigation

Resolution testing was performed for 62 discrepant results (38 affecting PPA, 24 affecting NPA). Of these, 34 discrepancies remained unresolved, including five with insufficient volume for additional testing (Table 4). After resolution testing, overall PPA improved to 95.3% (95% CI: 91.8–97.4%), while NPA remained at 99.9% (95% CI: 99.9–99.9%) (Table 5).

**TABLE 4:** Sample resolution testing^*a*^

Pathogen	QIAstat-Dx ME Panel	BFME panel comparator	PCR-BDS comparator	Culture SoC	Other SoC test	Discordance: PCR-BDS	Discordance: Simplexa HSV 1 & 2 and VZV direct kits	QIAstat-Dx ME result pre-discordance testing	Discordance investigation outcome
*E. coli* K1	Negative	Positive	N/A	N/A	N/A	Negative	N/A	FN	TN
	Negative	Positive	N/A	Negative	N/A	N/A	N/A	FN	TN
*H. influenzae*	Negative	Positive	N/A	Negative	N/A	Negative	N/A	FN	TN
	Positive	Negative	N/A	Negative	N/A	Negative	N/A	FP	FP
	Positive	Negative	N/A	Negative	N/A	Negative	N/A	FP	FP
	Positive	Negative	N/A	N/A	N/A	N/A	N/A	FP	NR
*L. monocytogenes*	Negative	Positive	N/A	Positive	LDT positive	N/A	N/A	FN	FN
*N. meningitidis*	Positive	Negative	N/A	Negative	N/A	Negative	N/A	FP	FP
*S. pneumoniae*	Positive	N/A	Negative	Negative	N/A	N/A	N/A	FP	FP
	Positive	N/A	Negative	Negative	N/A	N/A	N/A	FP	FP
	Positive	N/A	Negative	Negative	N/A	N/A	N/A	FP	FP
	Positive	N/A	Negative	Negative	N/A	N/A	N/A	FP	FP
	Positive	N/A	Negative	N/A	N/A	N/A	N/A	FP	NR
	Positive	N/A	Negative	Negative	N/A	N/A	N/A	FP	FP
EV	Negative	Positive	N/A	N/A	LDT negative	N/A	N/A	FN	TN
	Negative	Positive	N/A	N/A	N/A	Negative	N/A	FN	TN
	Positive	Negative	N/A	N/A	N/A	Negative	N/A	FP	FP
CMV	Negative	Positive	N/A	N/A	N/A	Negative	N/A	FN	TN
	Negative	Positive	N/A	N/A	N/A	Negative	N/A	FN	TN
	Positive	Negative	N/A	N/A	N/A	N/A	N/A	FP	NR
	Positive	Negative	N/A	N/A	N/A	Negative	N/A	FP	FP
	Positive	Negative	N/A	N/A	N/A	Negative	N/A	FP	FP
HSV-1	Negative	Positive	N/A	N/A	N/A	N/A	Not detected	FN	TN
	Negative	Positive	N/A	N/A	N/A	N/A	Not detected	FN	TN
HSV-2	Negative	Positive	N/A	N/A	N/A	N/A	Not detected	FN	TN
	Negative	Positive	N/A	N/A	Positive LDT for HSV-2	N/A	Detected (Ct = 38.9)	FN	FN
	Negative	Positive	N/A	N/A	Previous BFME panel positive	N/A	N/A	FN	NR
	Negative	Positive	N/A	N/A	N/A	N/A	Not detected	FN	TN
	Negative	Positive	N/A	N/A	N/A	N/A	Not detected	FN	TN
	Negative	Positive	N/A	N/A	N/A	N/A	Not detected	FN	TN
	Negative	Positive	N/A	N/A	N/A	N/A	Not detected	FN	TN
	Positive	Negative	N/A	N/A	N/A	N/A	Not detected	FP	FP
HPeV	Negative	Positive	N/A	N/A	N/A	N/A	N/A	FN	NR
	Negative	Positive	N/A	N/A	Previous BFME panel positive	N/A	N/A	FN	NR
	Negative	Positive	N/A	N/A	Positive LDT-HPeV	Negative	N/A	FN	TN
	Negative	Positive	N/A	N/A	N/A	Negative	N/A	FN	TN
	Positive	Negative	N/A	N/A	N/A	Negative	N/A	FP	FP
HHV-6	Negative	Positive	N/A	N/A	N/A	N/A	N/A	FN	NR
	Negative	Positive	N/A	N/A	N/A	Negative	N/A	FN	TN
	Negative	Positive	N/A	N/A	N/A	Negative	N/A	FN	TN
	Negative	Positive	N/A	N/A	N/A	Positive	N/A	FN	FN
	Negative	Positive	N/A	N/A	Allplex meningitis panel negative	N/A	N/A	FN	TN
	Positive	Negative	N/A	N/A	Previous BFME panel negative	Negative	N/A	FP	FP
	Positive	Negative	N/A	N/A	N/A	Positive	N/A	FP	TP
	Positive	Negative	N/A	N/A	N/A	Negative	N/A	FP	FP
	Positive	Negative	N/A	N/A	N/A	Negative	N/A	FP	FP
	Positive	Negative	N/A	N/A	N/A	Negative	N/A	FP	FP
	Positive	Negative	N/A	N/A	N/A	Negative	N/A	FP	FP
VZV	Negative	Positive	N/A	N/A	Previous BFME panel positive	N/A	Detected (Ct = 40)	FN	FN
	Negative	Positive	N/A	N/A	Previous BFME panel positive	N/A	Not detected	FN	TN
	Negative	Positive	N/A	N/A	Positive LDT-VZV	N/A	Not detected	FN	TN
	Negative	Positive	N/A	N/A	Positive LDT-VZV	N/A	Detected (Ct = 41)	FN	FN
	Negative	Positive	N/A	N/A	Positive LDT-VZV	N/A	N/A	FN	FN
	Negative	Positive	N/A	N/A	Positive LDT-VZV	N/A	Not detected	FN	TN
	Negative	Positive	N/A	N/A	Positive LDT-VZV	N/A	Not detected	FN	TN
	Negative	Positive	N/A	N/A	N/A	N/A	Not detected	FN	TN
	Negative	Positive	N/A	N/A	N/A	N/A	Detected (Ct = 40)	FN	FN
*C. gattii* / *C. neoformans* (not differentiated)	Negative	Positive	N/A	Negative	N/A	Negative	N/A	FN	TN
	Negative	Positive	N/A	Negative	CrAg negative	Negative	N/A	FN	TN
	Negative	Positive	N/A	N/A	CrAg positive	Negative	N/A	FN	TN
	Positive	Negative	N/A	N/A	N/A	Negative	N/A	FP	FP
	Positive	Negative	N/A	Negative	CrAg negative	N/A	N/A	FP	FP

^*a*^ BFME, BioFire FilmArray ME Panel; CMV, cytomegalovirus; CrAg, cryptococcal antigen; Ct, cycle threshold; EV, enterovirus; FN, false negative; FP, false positive; HHV, human herpesvirus; HPeV, human parechovirus; HSV, herpes simplex virus; LDT, laboratory-developed test; N/A, not applicable; PCR-BDS, polymerase chain reaction with bidirectional sequencing; SoC, standard of care; TN, true negative; VZV, varicella zoster virus; NR, discordance not resolved**.**

**TABLE 5:** PPA and NPA between the QIAstat-Dx ME Panel and comparator method for all clinical samples by target after resolution testing^*a*^

Pathogen	PPA			NPA		
	TP/TP + FN	%	95% CI	TN/TN + FP	%	95% CI
Bacteria						
*E.coli* K1	4/4	100.0	51.0–100.0	1,660/1,660	100.0	99.8–100.0
*H. influenzae*	10/10	100.0	72.2–100.0	1,651/1,654	99.8	99.5–99.9
*L. monocytogenes*	4/5	80.0	37.6–96.4	1,659/1,659	100.0	99.8–100.0
*M. pneumoniae*	0/0	N/A^*b*^	N/A	1,482/1,482	100.0	99.7–100.0
*N. meningitidis*	4/4	100.0	51.0–100.0	1,659/1,660	99.9	99.7–100.0
*S. agalactiae*	12/12	100.0	75.8–100.0	1,652/1,652	100.0	99.8–100.0
*S. pneumoniae*	12/12	100.0	75.8–100.0	1,463/1,469	99.6	99.1–99.8
*S. pyogenes*	0/0	N/A	N/A	1,401/1,401	100.0	99.7–100.0
Viruses						
CMV	3/3	100.0	43.9–100.0	1,658/1,661	99.8	99.5–99.9
EV	31/31	100.0	88.9–100.0	1,632/1,633	99.9	99.7–100.0
HSV-1	10/10	100.0	72.2–100.0	1,654/1,654	100.0	99.8–100.0
HSV-2	29/31	93.5	79.3–98.2	1,632/1,633	99.9	99.7–100.0
HPeV	4/6	66.7	30.0–90.3	1,657/1,658	99.9	99.7–100.0
HHV-6	26/28	92.9	77.4–98.0	1,631/1,636	99.7	99.3–99.9
VZV	62/66	93.9	85.4–97.6	1,598/1,598	100.0	99.8–100.0
Fungi and yeast						
*C. gattii* / *C. neoformans* (not differentiated)	12/12	100.0	75.8–100.0	1,650/1,652	99.9	99.6–100.0

^*a*^ CI, confidence interval; CMV, cytomegalovirus; EV, enterovirus; FN, false negative; FP, false positive; HHV, human herpesvirus; HPeV, human parechovirus; HSV, herpes simplex virus; NPA, negative percent agreement; PPA, positive percent agreement; TN, true negative; TP, true positive; VZV, varicella zoster virus.

^*b*^ N/A, not applicable.

### Co-detections

The QIAstat-Dx ME Panel identified co-detections in six samples, each with two pathogens: HHV-6 + *C. gattii*/*neoformans*, *S. agalactiae* + HHV-6, *S. pneumoniae* + HHV-6, *S. pneumoniae* + VZV, and HSV-2 + HHV-6 (two samples). The Ct values for co-detection cases are detailed in Table S3. Three out of six co-detection samples had at least one FP result.

## DISCUSSION

This multicenter study demonstrated that the QIAstat-Dx ME Panel achieves robust clinical performance for the detection of 16 pathogens typically associated with MEN/EN. The QIAstat-Dx ME Panel detected all assay targets and achieved high agreement with the comparator methods, with an overall PPA of 85.4% (222/260) and an NPA of 99.9% (25,712/25,736) across all clinical samples. After discrepancy resolution, PPA improved to 95.3%, supporting the QIAstat-Dx ME Panel as a reliable, fully automated multiplex PCR platform for rapid syndromic testing in suspected MEN/EN. While a small number of false positives occurred in the study, the QIAstat-Dx ME Panel demonstrated high analytical specificity, which is critical to avoid unnecessary treatment. Nonetheless, laboratory results should always be interpreted within the broader clinical context.

Multiplex PCR platforms like QIAstat-Dx allow simultaneous detection of multiple pathogens from a single CSF sample, enabling a more efficient diagnostic approach than conventional methods. In this study, *S. pneumoniae*, *H. influenzae*, *L. monocytogenes*, and *S. agalactiae* were the most frequently detected bacterial pathogens, reflecting well-known etiologies of bacterial MEN in Europe and the USA, with *S. agalactiae* being a leading cause in neonates (22, 23). Among viral pathogens, EV, HHV-6, HSV-2, and VZV were most common, broadly consistent with epidemiological patterns in viral MEN/EN across Europe (24). Co-detections were rare and detected in six samples, five of which harbored HHV-6, which was consistent with previous reports of herpesvirus reactivation in patients with MEN and potential detection of chromosomally integrated HHV-6 (25, 26).

While the BFME Panel remains the first and most widely adopted FDA-approved syndromic platform for CNS infections, and suitable performance has been seen in some studies (9–11, 27), concerns have emerged regarding reduced sensitivity for specific targets, such as HSV-1 (19) and *C. gattii*/*neoformans* (27–29). In our study, initial FN results for both HSV-1 and *C. gattii*/*neoformans* were resolved upon further DiaSorin or PCR-BDS discordance testing, suggesting that QIAstat-Dx performs reliably, even for these challenging analytes. However, cryptococcal antigen testing remains the preferred method for diagnosis of cryptococcal meningitis due to higher sensitivity than PCR tests, and one FN was positive for cryptococcal antigen (30). BFME was not employed as a comparator for *S. pneumoniae* because more false positives were observed than true positives in the BFME validation study, meaning an accurate PPA for the QIAstat-Dx ME Panel would not be established. Compared to PCR-BDS, the QIAstat-Dx ME Panel demonstrated better performance, identifying 12 true positives and six false positives.

Traditional diagnostic methods for MEN/EN, including CSF Gram stain, culture, antigen testing, serology, and neuroimaging (8), remain the standard of care, but are limited by long TAT, narrow pathogen coverage, and reduced sensitivity after antibiotic exposure. In contrast, the QIAstat-Dx ME Panel offers broad coverage of bacterial, viral, and fungal pathogens and delivers results in approximately 80 min, providing a timely and comprehensive diagnostic tool to inform clinical decision-making.

The QIAstat-Dx ME Panel provides further advantages over other multiplex platforms. It includes two additional targets not covered by BFME, *S. pyogenes* and *M. pneumoniae*, which is relevant given recent increases in invasive *S. pyogenes* infections in Europe and the USA during the coronavirus disease 2019 (COVID-19) pandemic (31–34), with incidence rates reaching 8.2–8.7 per 100,000 in 2022 (34, 35), although recent data suggest a return to typical seasonal trends (34, 36).

Moreover, the QIAstat-Dx platform provides amplification curves and Ct values, which may offer potential insights into pathogen load and clinical severity. Prior studies have correlated lower Ct values with worse outcomes in CNS infections and an increased risk of intensive care unit admission (37–39).

Several limitations of this study should be considered. For rare pathogens, the number of clinical positives was low, requiring supplementation with contrived CSF samples that may not fully mimic clinical complexity. Insufficient sample volume occasionally prevented repeat testing or discrepancy resolution. Comparator data were not available for all pathogens due to exclusions. The QIAstat-Dx ME Panel is also limited in scope of infection analysis. For example, it detects only *E. coli* K1 strains and encapsulated *N. meningitidis*, and it does not differentiate between *C. neoformans* and *C. gattii*. Nevertheless, it effectively targets the clinically relevant pathogens associated with MEN/EN (15).

Some samples for resolution testing underwent one or two freeze–thaw cycles, which could affect sample integrity, although prior data for the Simplexa HSV 1 & 2 assay showed no significant performance impact after two freeze–thaw cycles at −20°C (40), supporting the validity of our resolution testing approach.

### Conclusion

This multicenter study demonstrated that the QIAstat-Dx ME Panel delivers clinical performance comparable to the BFME Panel, supporting its validity as a reliable syndromic molecular diagnostic tool for MEN/EN. With its broad pathogen coverage, rapid TAT, and the ability to provide amplification curves and Ct values, the QIAstat-Dx ME Panel offers a valuable option for broad and timely detection of bacterial, viral, and fungal CNS pathogens. When used alongside the standard of care diagnostic methods, this panel has the potential to improve early diagnosis, guide targeted therapy, and support effective clinical management of patients with suspected MEN/EN.

### Summary

The QIAstat-Dx Meningitis/Encephalitis Panel demonstrated robust performance for detecting 16 clinically relevant central nervous system pathogens, supporting its use as a reliable, fully automated multiplex PCR platform for rapid syndromic testing in patients with suspected meningitis or encephalitis.

## Data Availability

The data supporting the findings of this study are available from the corresponding author on reasonable request.

## SUPPLEMENTAL MATERIAL

Supplemental tables and figure jcm.00738-26-s0001.docx Tables S1 to S3 and Fig. S1.

ASM does not own the copyrights to Supplemental Material that may be linked to, or accessed through, an article. The authors have granted ASM a non-exclusive, world-wide license to publish the Supplemental Material files. Please contact the corresponding author directly for reuse.
