# Supplementary material for: Multicenter evaluation of the multiplex polymerase chain reaction QIAstat-Dx Meningitis/Encephalitis Panel for the detection of central nervous system pathogens directly in the cerebrospinal fluid
Source: J Clin Microbiol. 2026 Oct 27;64(11):e00738-26. doi: 10.1128/jcm.00738-26 (PMC13753808; doi:10.1128/jcm.00738-26)
Supplement: Supplementary file 1 [file jcm.00738-26-s0001.docx]

**Supplementary Appendix**

*QIAstat-Dx ME Panel testing methods [15]*

CSF samples were collected and loaded manually into the QIAstat-Dx ME Panel Cartridge. A transfer pipette was used for sample transfer (200 μL) into the main port. Multiple steps were automatically performed in sequence using pneumatic pressure to transfer samples and fluids via the transfer chamber to their intended destinations. The extraction, amplification, and detection of nucleic acids in the sample were performed automatically by the QIAstat-Dx Analyzer 1.0:

- The sample was homogenized and cells were lysed in the lysis chamber of the QIAstat-Dx ME
- Nucleic acids were purified from the lysed sample via binding to a silica membrane in the purification chamber of the QIAstat-Dx ME Panel Cartridge in the presence of chaotropic salts and alcohol
- The purified nucleic acids were eluted from the membrane in the purification chamber and were mixed with the lyophilized PCR chemistry in the dried-chemistry chamber of the QIAstat-Dx ME Panel Cartridge
- The mixture of sample and PCR reagents was dispensed into the QIAstat-Dx ME Panel Cartridge PCR chambers, which contained lyophilized assay-specific primers and probes
- The QIAstat-Dx Analyzer 1.0 was used to create the optimal temperature profiles to carry out effective multiplex real-time PCR and perform real-time fluorescence measurements to generate amplification curves
- The QIAstat-Dx Analyzer 1.0 Software was used to interpret the resulting data and process controls and deliver a test report.
- Note although study was conducted with the QIAstat-Dx Analyzer 1.0, this analyzer is functionally the same as QIAstat-Dx Analyzer 2.0, providing identical results, although, the 2.0 version has updated software. Therefore, data generated on Analyzer 1.0 is applicable to Analyzer 2.0 users.

**Supplementary Table S1.** Pathogen Overview Including Classification for Each Pathogen Detected by the QIAstat-Dx ME Panel and LoD, with Biofire LoD for comparison

| **Pathogen** | **Classification** | **QIAstat-Dx LoD (units)** | **Biofire LoD (units)** |
| --- | --- | --- | --- |
| *E. coli K1* | Bacterium (DNA) | 7.86E+02 (CFU/ml) | 1.0E+03 (CFU /mL) |
| *H. influenzae* | Bacterium (DNA) | 2.54E+03 (CFU/ml) | 1.0E+03 (CFU/mL) |
| *L. monocytogenes* | Bacterium (DNA) | 2.10E+04 (CFU/ml) | 1.0E+03 (CFU/mL) |
| *N. meningitidis* (encapsulated) | Bacterium (DNA) | 1.33E+01 (CFU/ml) | 1.0E+02 (CFU/mL  (~ 1.80E+03 copies/mL)) |
| *S. agalactiae* | Bacterium (DNA) | 3.38E+03 (CFU/ml) | 1.0E+03 (CFU/mL) |
| *S. pneumoniae* | Bacterium (DNA) | 7.14E+02 (CFU/ml) | 1.0E+02 cells/mL ((~1.50E+03 copies/mL)) |
| *S. pyogenes* | Bacterium (DNA) | 1.80E+03 (CFU/ml) | N/A |
| *M. pneumoniae* | Bacterium (DNA) | 9.99E+01 (CCU/ml) | N/A |
| HSV-1 | Herpesvirus (DNA) | 3.38E+02 (TCID_50_/mL) | 2.5E+02 TCID_50_/mL ((1.51E+03 copies/mL or IU/mL)) |
| HSV-2 | Herpesvirus (DNA) | 2.81E+01 (TCID_50_/mL) | 5.0E+01 (TCID_50_/mL (1.29E+03 copies/mL)) |
| HHV-6 | Herpesvirus (DNA) | 7.29E+04 (copies/mL) | 1.0E+04 (copies/mL or IU/mL) |
| EV | Picornavirus (RNA) | 5.06E+02 (TCID_50_/mL) | 5.0E+01 (TCID_50_/mL) |
| HPeV | Picornavirus (RNA) | 1.07E+03 (TCID_50_/mL) | 5.0E+02 (TCID_50_/mL) |
| VZV | Herpesvirus (DNA) | 1.71E+03 (copies/mL) | 1.0E-01 (TCID_50_/mL (1.66E+03 copies/mL)) |
| CMV | Herpesvirus (DNA) | 1.00 E+01 (TCID_50_/mL) | 1.0E+02 (TCID_50_/mL (4.30E+03 copies/mL or IU/mL)) |
| *C. gattii/ C. neoformans*  (not differentiated) | Fungal (yeast) (DNA) | 1.32E+04  2.21E+03 (CFU/mL) | 1.0E+02 (CFU/mL) |

Abbreviations: LoD, Limit of Detection, CMV, cytomegalovirus; EV, enterovirus; HHV, human herpesvirus; HPeV, human parechovirus; HSV, human simplex virus; TCID, tissue culture infectious dose; VZV, varicella zoster virus.

**Supplementary Table S2.** Composition of External Control Mixes

| Mix | Pathogen |
| --- | --- |
| 1 | *S. pneumoniae* |
|  | *N. meningitidis* |
|  | *S. agalactiae* |
|  | *L. monocytogenes* |
|  | *H. influenzae* |
|  | EV |
| 2 | HSV-1 |
|  | CMV |
|  | HHV-6 |
|  | *S. pyogenes* |
|  | VZV |
| 3 | *M. pneumoniae* |
|  | *C. gattii* |
|  | HPeV |
|  | HSV-2 |
|  | *E. coli* K1 |
| 4 | Negative – no pathogens |

Abbreviations: CMV, cytomegalovirus; EV, enterovirus; HHV, human herpesvirus; HPeV, human parechovirus; HSV, herpes simplex virus; VZV, varicella zoster virus.

**Supplementary Table S3.** Ct Values of Clinical Samples Where Co-Detections Were Identified as per the QIAstat-Dx ME Panel

|  | Samples with co-detections identified by the QIAstat-Dx ME Panel (n = 6) |  |  |  |
| --- | --- | --- | --- | --- |
| Sample | Pathogen 1 | Ct 1 | Pathogen 2 | Ct 2 |
| 1 | HSV-2 | 32.7 | HHV-6 | 26.3 |
| 2 | HSV-2 | 32 | HHV-6^a^ | 36.3 |
| 3 | *C. gattii / C. neoformans^b^* (not differentiated) | 35.3 | HHV-6^c^ | 36.7 |
| 4 | *S. agalactiae* | 25.1 | HHV-6 | 24.2 |
| 5 | *S. pneumoniae* | 15.7 | HHV-6 | 26.3 |
| 6 | *S. pneumoniae^d^* | 37.9 | VZV | 29.6 |

BDS, bidirectional sequencing; Ct, cycle threshold; FP, false positive; HSV, herpes simplex virus; PCR, polymerase chain reaction; SoC, standard of care; VZV, varicella zoster virus.

^a^SoC negative, PCR/BDS negative, considered FP.

^b^Fungal culture negative, antigen test negative, BioFire FilmArray ME panel negative, no volume for PCR/BDS, considered FP.

^c^PCR/BDS negative, considered FP.

^d^Negative by culture, PCR/BDS negative, considered FP.

**Supplementary Figure S1**. Ct values for all pathogens as per the QIAstat-Dx ME Panel.

Box and Whisker plot showing Median and Inter-Quartile Range. X = mean value.

Abbreviations: CMV, cytomegalovirus; Ct, cycle threshold; HHV, human herpesvirus; HPeV, human parechovirus; HSV, herpes simplex virus; VZV, varicella zoster virus.

^a^Not differentiated.
